# Maturation capacity, morphology and morphometric assessment of human immature oocytes after vitrification and in-vitro maturation

**Published:** 2011

**Authors:** Saeedeh Nazari, Mohammad Ali Khalili, Forouzan Esmaielzadeh, Mehdi Mohsenzadeh

**Affiliations:** 1 Researh and Clinical Center for Infertility, Shahid Sadoughi University of Medical Sciences, Yazd, Iran.; 2 Department of Animal Sciences, Marvdasht Branch, Islamic Azad University, Marvdasht, Iran.; 3 Shiraz University of Medical Sciences, Shiraz, Iran.

**Keywords:** *IVM*, *Oocyte*, *Vitrification*, *Maturation*, *Morphology*, *Morphometry*

## Abstract

**Background:**

In general, 15% of oocytes collected in ART cycles are immature. These oocytes may be cryopreserved further for use in in-vitro maturation (IVM) program.

**Objective:**

The aim of this study was to determine maturation capacity, morphometric parameters and morphology of human immature oocytes in both fresh IVM (fIVM) and vitrified-IVM (vIVM) oocytes.

**Materials and Methods:**

93 women who underwent controlled ovarian stimulation for ART were included. The immature oocytes (n=203) were divided into two groups: the first group (n=101) directly matured in vitro; and the second group (n=102) first vitrified, then matured in vitro. All oocytes underwent IVM in Ham’s F10 supplemented with LH+FSH and human follicular fluid. After 48h of incubation, the oocyte maturation rates, as well as morphometric and morphologic characteristics were assessed using cornus imaging and were compared.

**Results:**

Oocyte maturation rates were reduced in vIVM, (40.4%), in comparison with fIVM (59.4%, p<0.001). Following morphometric assessment, there was no difference in the mean oocyte diameters (µm) between fIVM and vIVM, 156.3±6.8 and 154.07±9.9, respectively. Other parameters of perimeters, egg areas, as well as oocyte and ooplasm volumes were similar in two groups. In addition, more morphologic abnormalities, such as, vacuole, and dark oocyte were observed in vIVM oocytes.

**Conclusion:**

fIVM was more successful than vIVM groups. No statistical differences were noticed in morphometry assessment in two groups. This suggests that morphometric parameters can not be applied as prognosis factor in oocyte maturation outcome in IVM program.

## Introduction

In-vitro maturation (IVM) and cryopreservation of immature oocytes has been proposed as an alternative for conventional IVF treatment. IVM has several advantages of reducing the costs, avoidance of the side effects of ovarian hyper stimulation syndrome (OHSS) and simplified treatment for certain infertile couples (1-3). Currently, most IVM protocols have supplemented with FSH and/or LH into the culture medium for oocyte maturation (1). Cryopreservation could benefit to patients who have specific situation such as chemotherapy or radiation (4). Several factors affect the survival and viability of human oocytes and embryos such as exposure time of cells to different cryoprotectant solutions, their different concentrations and rate of ice crystal formation (3). One established method for the cryopreservation of human oocyte and embryo is vitrification technology (3, 5). During recent years, studies have shown that vitrification offers new interesting perspectives in the field of oocyte cryopreservation, demonstrating less traumatic than slow cooling (6, 7).

After ovarian stimulation, approximately 15% of oocytes are immature at the GV and M1 stages. In 1991, the first human birth resulting from IVM was reported by Cha and associates (8). In 1994, Trounson *et al* reported the first birth from untreated polycystic ovarian (PCO) patient (9). In many species, it has been demonstrated that a suitable oocyte size is necessary for the continuation of meiosis and maturation. Durinzi *et al* (1995) demonstrated that the oocyte diameter at the time of retrieval from un-stimulated cycles has affected IVM in human oocytes to resume meiosis and completion of their maturation (10).

In addition, Mikkelsen and Lindenbery (2001) demonstrated that there was no difference in morphology between the in-vitro matured oocytes in normal ovaries and polycystic ovaries (11). In 1993, Albert *et al *reported that the diameter of a normal human oocytes was 60-150µm (12). Recently, Lasiene *et al* (2009) evaluated the size of the zona pellucida (ZP) to vary from 10 to 31 µm, which was not related to the cytoplasm diameter (13). Bao *et al *(2000) showed that developmental changes occurring during the final stages of oocyte growth are critical for full developmental competence (14). 

Also, immature oocytes that underwent IVM were smaller than in-vivo matured oocytes. Therefore, the oocyte diameter grown in culture are affected on maturation (15). Michelmman *et al* (1995) examined micro morphometry and sperm binding patterns between fertilized and unfertilized human oocytes after in vitro fertilization. They showed that there were no significant differences in morphometry between fertilized and unfertilized oocytes. 

Though, differences demonstrated in the size of perivetilline space and the ZP (16). Durizi *et al* (1995) showed the size of human oocytes depend with ability to resume meiosis and complete maturation in IVM with un-stimulated cycles (10). Although, there is some evidence available about morphometry and morphology in human oocytes, but there is no report on morphology and morphometry of human immature oocytes following fresh-IVM and vitrified-IVM in ART cycles. Therefore, the main objective of this prospective study was to investigate the success of cryopreservation of immature human oocytes using vitrification. This was followed by comparing oocyte maturation rates, as well as morphology and morphometry assessments. 

## Materials and methods

**Patients**

A total of 93 infertile women were included in this cross-sectional study. The investigation took place over a period of 5 months in 2010 at Research and Clinical Center for Infertility in Yazd. This study was approved by ethics committee of our institution. Oocytes were allocated in two groups of fresh-IVM (fIVM; n=101) and vitrified-IVM (vIVM; n=102). 

**Oocyte collection**

The oocyte collection was performed 36h after 10,000 IU of hCG (IBSA Co, Switzerland) injection. Transvaginal ultrasound was used for oocytes collection with a single lumen aspiration needle (Wallace, Smiths Medical International, UK) with a reduced pressure of 150 mmhg. The collected oocytes were assessed for nuclear maturity under the stereo microscope (Olympus Co, Japan). After denudation with 80 IU hyaluronidase (Sigma Co, USA) and mechanical pippetting, the oocytes were assessed for maturity.The oocytes that extruded the first polar body were considered mature (metaphase II) and were inseminated using intracytoplasmic sperm injection (ICSI) technique.

**Follicular fluid preparation**

Preparation of human follicular fluid (HFF) was performed according to the method described previously (17). HFF was obtained from women who underwent follicular puncture. HFF was centrifuged at 3500 RPM for 10 minutes. Blood and granulosa cells were settled, and pure HFF was transferred to new tube. The HFF was then inactivated in water bath at 56°C for 30 min. At last, HFF was filtered with 0.22 µm filters, then aliquoted and stored at -20°C before use.

**In vitro maturation**

Immature oocytes were denuded of Cumulus-oocyte complexes (COCs). The oocytes were then washed in 3 drops of IVM medium and were cultured in IVM medium containing Ham’s F10 (Biochrom Co, Germany) supplemented with 0.75 IU LH, 0.75 IU FSH (Ferring Co, Germany)with 40% HFF at 37°C in an incubator with 5% CO_2 _and 95% air with high humidity (98%). Oocytes were observed under an inverted microscope (Nikon Co, Japan) after 48h to determine maturity.

**Vitrification of immature oocytes**

Immature oocytes were frozen using a modified vitrification method reported by Al-Hasani *et al* (2007) (3). Initially, immature oocytes were placed in an equilibration solution containing 7.5% ethylene glycol (EG) (Merck Co, Germany), 7.5% dimethyl sulphoxide (DMSO) (Merck Co, Germany) in Ham’s F10 media supplemented with 20% human serum albumin (HSA) (Plasbumin Co, USA) for 5-15 min at room temperature. Then, oocytes were removed and placed into vitrification solution containing 15% EG, 15% DMSO and 0.5M socruse (Sigma Co, USA) in Ham’s F10 medium supplemented with 20% HSA for 50-60 Sec at room temperature. After this stage, the oocytes were loaded into cryotops that were plunged into liquid nitrogen quickly. Next, the cap was placed on the cryotop and put into the cane. Finally, the samples were transferred to the liquid nitrogen storage tank for 2 months.

**Thawing of immature oocytes**

Thawing of the oocytes was performed by placing the cryotop in thawing solution in five stages: thawing solution (Ham’s F10 supplemented with 20% HSA and 1M sucrose) for 50-60 Sec, dilution solution 1 (Ham’s F10 supplemented with 20% HSA and 0.5M sucrose) for 3 min, dilution solution 2 (Ham’s F10 supplemented with 20% HSA and 0.25 M sucrose) for 3 min, washing solution 1 and 2 (Ham’s F10 supplemented with 20% HSA) each for 3-5 min. After this stage, the oocytes were placed in IVM medium for 48h in incubator. 

Viability of vitrified oocytes was evaluated microscopically 2-3 h after culture, based on the morphology of cytoplasm (18). The dead oocytes were shown with broken membrane and dark ooplasm.

**Morphometry and morphology evaluations**

After a culture period of 48h, all oocytes were assessed for maturity under an inverted microscope; Matured oocytes were evaluated for morphology and morphometry. The matured oocytes were divided into two groups (Group 1=fIVM, Group 2=vIVM). Measurements accomplished with inverted microscope equipped with cornus imaging program (Research instruments Ltd Co, UK). Measurements comprised of the diameters of whole oocyte (µm), ooplasm (µm), width of ZP (µm), perimeters and areas of whole oocyte and ooplasm (µm^2^), and volume of whole oocyte and ooplasm (µm^3^) (Figure 2). 

To calculate the average diameter of different parts of each oocyte comprising of whole oocyte, ooplasm and ZP were calculated in 4 different parts. Also, the morphology of each mature oocyte was determined by characteristics of refractile body (RF), granularity, vacuole, smooth endoplasmic reticulum (SER), bull’s eye, ZP and perivite line space (PVS), coloration of ZP and ooplasm (dark or normal), polar body shape (normal or fragmented), oocyte shape (circular or irregular) and PVS debris in both groups (Figure3).

**Statistical analysis**

Statistical analysis was carried out using t-test for morphometrical data and χ^2^for morphological data by SPSS (version 16). p<0.05 was considered significant.

## Results

**Figure 1:**
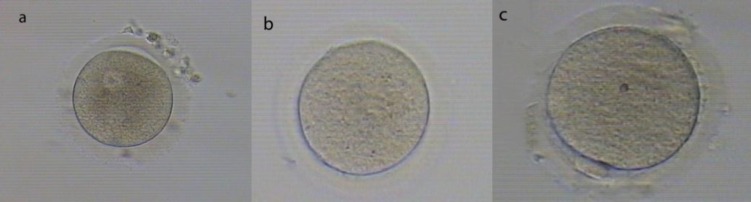
Morphological markers characterizing the meiotic status of human oocytes.

As shown in table I, there were no significant differences in characteristics of age, etiology of infertility, total number of retrieved oocytes and oocytes stage (GV, MI) between fIVM with vIVM groups. The numbers of immature oocytes (Figure 1) were; GV=72, MI=29 in fIVM and GV=66, MI=36 in vIVM group. There were no significant differences in the numbers of GV and MI oocytes in fIVM and vIVM.

The oocyte survival rate was 87.25% (89/102) when the oocytes were vitrified. The oocyte maturation rate was 40.4% (36/89) when the oocytes were vitrified and then underwent IVM after warming. This was significantly lower (p<0.001) than the immature oocytes matured in vitro without vitrification which was 59.4% (61/101) (Table II). Also, a total of 16 immature oocytes were collected from a patient with OHSS. However, the rate of maturity after IVM protocol was as low as 12.5%. 

The data also showed that, morphometric and morphologic analyses of matured oocytes were possible in 96 oocytes (60 fIVM, 36 vIVM). Although, there were no significant differences in the diameters, areas, perimeters and volumes between the oocytes vitrified at the immature stage than those obtained using the oocytes without vitrification for IVM (Table III). As shown in table IV, there were no significant differences between RF, fragmented polar body, regularity of shape, granularity, PVS debris and width of PVS in fIVM and vIVM groups. However, in vIVM oocytes, the rates of vacuoles and dark oocytes were significantly higher than fIVM group. Both groups didn’t show any SER and dark ZP in oocytes that were matured in-vitro. In fIVM, the most common morphological abnormality was presence of RF.

**Table I:** Characteristics of patients in fresh IVM and vitrified-IVM groups

**Variables**	**Fresh-IVM (n=101)** **(GV=72,MI=29)**	**Vitrified-IVM (n=102)** **(GV=66,MI=36)**	**p-value**
Age (years) (mean±SD)	29.3±5.9	32.2±5.5	NS
Female factor infertility	43(50.6)	42(49.4)	0.920
Male factor infertility	48(50.0)	48(50.0)	0.910
Both(male and female factors infertility)	10(45.5)	12(54.5)	0.724

NS= not significant

Values inside parentheses represent (%).

**Table II:** Comparison of maturation rates of human oocytes in two groups of IVM

**Variables**	**Fresh IVM (n=101)**	**Vitrified-IVM (n=89)**	**p-value**
MII oocyte (matured)	60(59.4)	36(40.4)	<0.001*
oocyte arrest	36(35.6)	14(15.7)	-
Degeneration	3(3)	38(42.7)	<0.001*
Parthenogenesis	2(2)	1(1.2)	<0.001*

Values inside parentheses represents (%).

* indicates significant difference between fresh IVM and vitrified-IVM

**Table III:** MII oocyte morphometric comparison between fresh IVM and vitrification groups in all in-vitro matured oocytes (n=96).

**Variables**	**Fresh-IVM (n=60)**	**Vitrified-IVM (n=36)**	**Unit**	**p value**
Oocyte diameter	156.3 ± 6.8	154.07 ± 9.9	µm	0.183
Ooplasm diameter	115.3 ± 4.6	114.09 ± 7.5	µm	0.306
Zona pellucida width	17.8 ± 3.2	17.3 ± 2.6	µm	0.342
Oocyte area	188.8×10^2 ^± 3.1×10^3^	234.3×10^2^ ± 2.9×10^4^	µm^2^	0.353
Oocyte perimeter	4.9×10^2^ ± 2.9×10	4.8×10^2^ ± 3.1×10	µm^2^	0.343
Ooplasm area	1.07×10^4^ ± 1.5×10^3^	1.02×10^4^ ± 1.6×10^3^	µm^2^	0.196
Ooplasm perimeter	3×10^2^ ± 2.3×10	3.6×10^2^ ± 2.4×10	µm^2^	0.273
Oocyte volume	2×10^6 ^± 2.7×10^5^	1.9×10^6^ ± 4.1×10^5^	µm^3^	0.293
Ooplasm volume	1×10^6 ^± 2.7×10^5^	0.87×10^6^ ± 0.58×10^6^	µm^3^	0.373

Values are mean ± SD.

**Table IV:** MII oocyte morphologic comparisons between the two IVM groups

**Variables **	**Fresh IVM (n=60)**	**Vitrified-IVM (n=36)**	**p-value**
Refractile body	37(60.7)	20(55.6)	0.673
Fragmented polar body	28(45.9)	19(52.8)	0.535
Regular shape	49(80.3)	30(83.3)	0.792
Ooplasm granularity	12(19.7)	11(30.6)	0.323
Vacuole	4(6.6)	9(25.0)	<0.01*
Smooth endoplasmic reticulum	0(0)	0(0)	-
PVS debris	7(11.5)	7(19.4)	0.371
Dark zona pellucida	0(0)	0(0)	-
Dark oocyte	2(3.3)	11(30.6)	<0.001*
Bull’s eye	5(8.2)	0(0)	0.154
Width zona pellucida (%)	3(4.9)	0(0)	0.293
Width PVS	9(14.8)	4(11.1)	0.762

NS= not significant.

Values inside parentheses represents (%).

* indicates significant difference between fresh IVM and vitrified-IVM.

a) Immature germinal vesicle (GV) oocyte.

b) Immature germinal vesicle breakdown (GVBD) oocyte (M1).

c) Mature oocyte (MII) .

**Figure 2:**
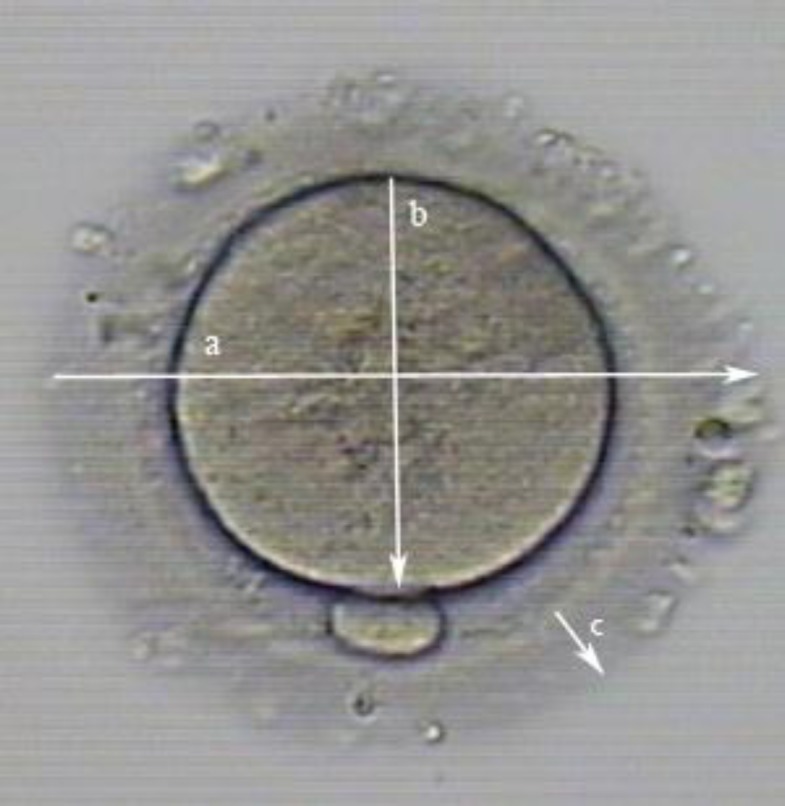
Dimentions that were measured during mormorphometry evaluation of oocytes after IVM.

a) Diameter of oocyte

b) Diameter of ooplasm

c) Diameter of ZP

**Figure 3:**
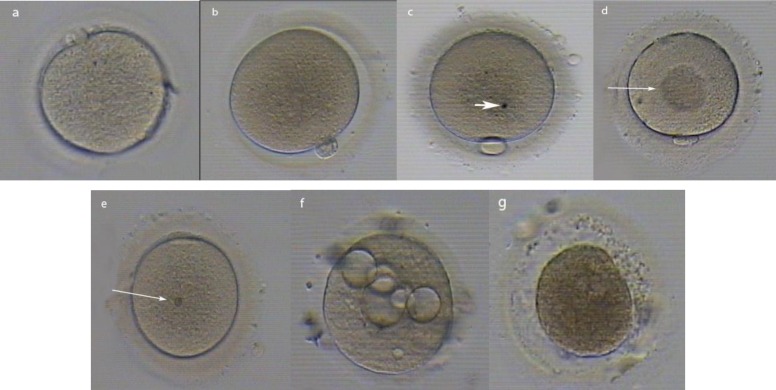
Various morphological abnormalities exhibited by MII oocytes.

a) Fragmented polar body observed in vitrified-IVM group.

b) Wide PVS without debris in one matured oocyte after fresh IVM.

c) A typical refractile body within ooplasm.

d) Central granularity in human oocytes after IVM.

e) Bull’s eye within ooplasm.

f) Several large vacuoles in oocyte.

g) Degenerated oocyte after vitrification.

## Discussion

Microtubular spindle in MII oocyte is sensitive to low temperature and cryopreservation. However, GV oocytes with chromosomes within the nuclear membrane are protected from these disorders (19). We evaluated morphology and morphometry of oocytes after maturation in fIVM and vIVM in patients undergoing ART program.

The aim was to found out whether differences existed in the morphology and morphometry of human oocytes after IVM in two groups. In this study, the maturity after IVM was demonstrated to be higher in fIVM when compared with vIVM. Also, the results showed that the rates of GV and M1 arrest in fIVM were higher than vIVM. However, the percentage of maturity (MII) was higher in fIVM, when compared with vIVM. In addition, rates of degeneration were significantly increased in vIVM group. Therefore, immature oocytes in ART cases should immediately undergo the IVM technology, though they may be cryopreserved successfully if it becomes necessary.

According to Kim *et al* (2000), almost 78.5% of the immature oocytes retrieved in stimulated cycles were matured, and 12.3% of oocytes remained at the GV stage (17). Russel (1998) showed that maturation rate was 63% in naked and compact oocytes in unstimulated cycles which is similar to our results (20). Later, Toth *et al* (1994) demonstrated that 83.3% of the oocytes were matured after thawing of cryopreserved oocytes (21). 

Recently, Cao *et al* (2009) reported the survival rates of human immature oocytes in vitrified oocytes as high as 85.4% after warming. This survival rate was similar to our study. For this reason, it seems that high survival rate can be obtained when human oocytes are vitrified at the immature stage. Also, Liu (2010) reported that, the removal of granulosa cells would affect IVM of immature oocytes (2).

In this study, the rates of maturation in vitrified oocytes were somewhat similar to study done by Cao *et al* (2009). But, rate of maturity following IVM of immature oocytes without vitrification were lower when compared to above (18). In addition, morphometric comparison on fIVM and vIVM showed only minor differences between two groups. During growth phase, oocyte diameter increases from 30 to 110 µm over a period of at least 8 weeks in human (22). When oocytes are matured in-vivo, the size of oocyte normality is approximately 110-120 µm, excluding the ZP, also whole size of oocyte was almost 150 µm (23).

 We found that when immature oocytes were vitrified and then matured using IVM technology, no differences were noticed in parameters. This evidence showed that vitrification seems to have no effect on morphometric parameters when compared with fresh IVM. Bertand *et al *(1995) reported that human ZP varies from 10-30 µm with a mean of 17.5 µm (24). 

Cavilla (2008) found zona diameter from the oocytes matured in vitro and then fertilized were larger than diameters of the in-vivo matured oocytes (12). In present study, all mature oocytes had a ZP thickness of 17.8 and 17.3 µm in fIVM and vIVM groups, respectively.

This was almost similar to the data reported by Cavelia *et al *(15). Also, the mean oocyte diameter in our study was statistically higher to the value obtained by Salata and associates (23). This difference could be credited to the large sample size evaluated by Salata *et al *(23). In addition, there were no significant differences in volume, perimeter, area ooplasm and oocyte between fIVM and vIVM. Michelman *et al* (1995) also found the volume of human oocytes to be similar to our results. 

In addition, most of abnormalities were noticed in the oocytes that underwent IVM technology. This study demonstrated that rates of vacuoalization and dark oocytes were significantly increased in vIVM than fIVM. This may prove that oocyte cryopreservation cause some irreversible structural damage. Lasiene *et al* (2000) reported the quality of oocytes deliberated by the structure of COC.This procedure gives us some information about the quality of oocytes(13). 

Also, they showed that oocytes with less cumulus compact, deficiency granularity and dark ooplasms have higher developmental competence, and the quality is increased when oocytes covered by more layers of cells (25, 26). According to defects in oocytes, they are categorized into single, double, multiple and nodefects. About this classification, no one reported any differences in fIVM and vIVM groups. Balaban *et al* (2006) classified oocytes abnormalities, that were similar to our study (27). Xia (1997) showed that oocyte with fragmented polar body have a lower fertilization than normal polar body. Also, oocytes with extensive and fragmented polar body have a worse developmental after fertilization (28). 

Ubalidi *et al* (2008) evaluated morphology of oocytes and reported that such as fragmented polar body and large PVS are rather higher than other abnormalities (49% and 32%), respectively (29). Our results showed that the rates of fragmented polar body and SER are approximality similar to theirs. Other defects, such as Bull’s eye, thick ZP, SER and dark ZP were not observed in oocytes under investigation.

## Conclusion

The rates of human oocytes recovery following vitrification concomitant with IVM program are acceptable. However, morphometrical assessment of oocyte does not seem to play a role in prognosis of oocyte maturation outcome.
